# Cannabidiol as Modulator of Spontaneous Adipogenesis in Human Adipose-Derived Stem Cells

**DOI:** 10.3390/molecules30112367

**Published:** 2025-05-29

**Authors:** Giovannamaria Petrocelli, Luca Pampanella, Provvidenza Maria Abruzzo, Sara Cruciani, Carlo Ventura, Silvia Canaider, Federica Facchin

**Affiliations:** 1 Department of Medical and Surgical Sciences (DIMEC), University of Bologna, Via Massarenti 9, 40138 Bologna, Italy; giovannam.petrocell2@unibo.it (G.P.); luca.pampanella2@unibo.it (L.P.); provvidenza.abruzzo2@unibo.it (P.M.A.); carlo.ventura@unibo.it (C.V.); federica.facchin2@unibo.it (F.F.); 2 Department of Biomedical Sciences, University of Sassari, Viale San Pietro 43/B, 07100 Sassari, Italy; scruciani@uniss.it; 3 National Laboratory of Molecular Biology and Stem Cell Bioengineering of the National Institute of Biostructures and Biosystems (NIBB) c/o Eldor Lab, Via Corticella 183, 40129 Bologna, Italy; 4 IRCCS Azienda Ospedaliero-Universitaria di Bologna, Via Massarenti 9, 40138 Bologna, Italy

**Keywords:** cannabidiol, adipose-derived stem cells, adipogenesis, in vitro assays, peroxisome proliferator-activated receptor gamma, CCAAT/enhancer-binding protein alpha, fatty acid-binding protein 4

## Abstract

Mesenchymal stem cells isolated from human adipose tissue (hASCs) are a promising tool for tissue repair due to their ability to differentiate into specific cell lineages. The possibility of modulating the adipogenic differentiation of hASCs is crucial in improving their therapeutic potential. This study aimed to investigate the effects of cannabidiol (CBD), a phytocannabinoid isolated from *Cannabis sativa* L., on hASCs. Few studies have evaluated its role in stem cell (SC) properties and their differentiation potential. hASCs were first treated with different concentrations of CBD (ranging from 0.1 to 10 μM) to assess its effects on viability, demonstrating that this molecule is non-toxic, except at the concentration of 10 μM. Subsequently, the role of CBD in the proliferation, metabolism and adipogenic potential of hASCs was analyzed. CBD promoted adipogenesis in a dose-dependent manner, even in the absence of differentiation medium. This result was evidenced by the presence of lipid vacuoles, the expression of adipogenic markers, cytoskeletal actin rearrangement and modulation in the expression of osteogenic genes. Although the results indicated a role of CBD in promoting hASC adipogenesis, further research will be needed to explore the mechanism of action of CBD in SC differentiation and to deepen its utility in SC-based approaches.

## 1. Introduction

Regenerative medicine is an emerging field of medical research that aims to repair, replace or regenerate tissues and organs damaged by injuries, traumas, neoplasms and degenerative diseases [1]. Stem cells (SCs), with their properties of self-renewal and multilineage differentiation, represent a fundamental tool of cell-based therapy for the regeneration of compromised body districts [2]. Among mesenchymal stem cells (MSCs), those derived from human adipose tissue (hASCs) have multiple advantageous characteristics. Their harvesting is simpler and less invasive compared to other tissues, such as bone marrow; there are few ethical limitations in their use; and they show a lower risk of serious complications in transplantation [3,4].

The modulation of the SC differentiation process is one of the goals in the regenerative medicine field [5]. In fact, these cells, once differentiated, can replace lost or damaged cells within a tissue to restore its integrity and function. In this context, the ability of hASCs to differentiate into adipocytes is crucial for their therapeutic applications, especially in the reconstruction of soft tissue characterized by deficits in the subcutaneous adipose layer caused by trauma, tumor resections, congenital abnormalities or diabetic ulcers [5].

Adipogenesis is a complex process that leads to the differentiation of SCs, involving multiple signaling pathways, transcription factors and genes [6]. To induce this process in vitro, a specific culture medium is commonly used. However, some studies have shown that hASCs long maintained in standard medium (SM) can spontaneously differentiate into mature adipocytes [7]. Moreover, several chemical and physical agents have shown the ability to modulate ASC differentiation potential [8].

Among them, cannabidiol (CBD), a phytocannabinoid derived from *Cannabis sativa* L., first isolated in 1940 [9], has attracted increasing interest in recent years due to its therapeutic effects [10,11]. Preclinical and clinical research has demonstrated multiple properties of CBD, including antioxidant, anti-inflammatory, immunomodulatory, anticonvulsant, neuroprotective, anxiolytic and antipsychotic activities [12,13]. Therefore, CBD can be considered a “well-being” molecule capable of promoting human health. In particular, CBD acts by counteracting oxidative stress damage associated with aging or diseases, as well as playing a crucial protective role at the nervous-system level [11,12]. Based on these findings, some authors have studied the role of CBD with respect to the biological properties of SCs to assess whether this molecule could be used in vitro and/or in vivo to improve the potential of SCs and, consequently, their therapeutic efficacy in regenerative medicine approaches. Published studies have focused mainly on the role of CBD in SC proliferation, migration and differentiation into osteogenic, neurogenic and adipogenic lineages [14,15,16,17,18,19], demonstrating the ability of CBD to promote these properties of SCs, as well as to protect them from apoptosis, inflammation and oxidative stress [20]. Moreover, the involvement of CBD in multiple molecular pathways highlights its complex physiological activity [12].

Phytocannabinoids—especially CBD—can influence the development of adipose tissue by regulating the activation of *peroxisome proliferator-activated receptor gamma* (*PPARγ*), a modulator of adipocyte differentiation and lipid storage [16,18]. In fact, CBD promotes the adipogenesis of both human and mouse MSCs (derived from adipose tissue and bone marrow), acting as a moderate PPARγ activator and thereby increasing the expression of key genes such as *CCAAT/enhancer-binding protein alpha* (*CEBPα*) and *fatty acid-binding protein 4* (*FABP4*), both of which are involved in the adipogenic process [16,18].

Most studies that have investigated the role of CBD in SC differentiation were performed using a specific induction medium (IM). Instead, this research aimed to evaluate the effect of CBD on the main biological properties of hASCs, including differentiation potential, by adding the molecule directly to the SM. The intrinsic ability of CBD to modulate hASC adipogenesis could have great potential in SC-based approaches that involve the transplantation of pre-conditioned or directly conditioned SCs into a biological niche. Therefore, after evaluating the cytotoxicity of different concentrations of CBD (spanning from 0.1 to 10 μM) on hASCs, proliferation and metabolic activity were investigated in hASCs treated with CBD for 72 hours (h). Subsequently, the adipogenic potential of CBD was studied in CBD-treated hASCs cultured in the absence of pro-adipogenic IM by analyzing the cell morphology, the formation of lipid droplets, the rearrangement of F-actin filaments and the expression of specific adipogenic and osteogenic markers.

## 2. Results

### 2.1. Characterization of hASCs

**Figure 1:**
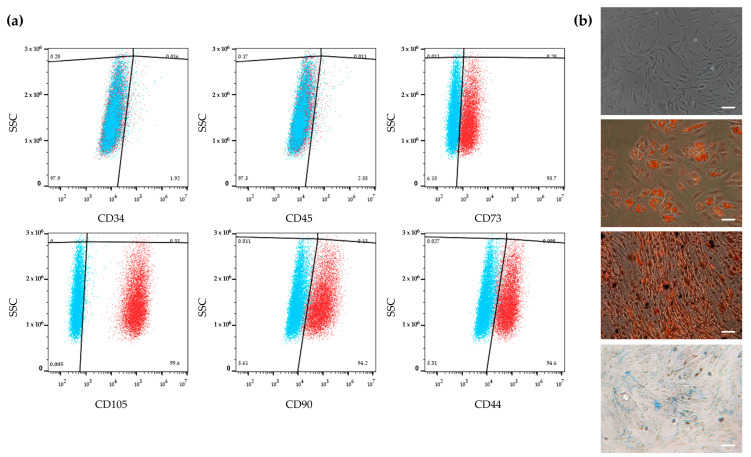
Characterization of human adipose-derived stem cells (hASCs). (**a**) Immunophenotypic analysis of hASCs by flow cytometry. hASCs are negative for the expression of CD34 and CD45 hematopoietic surface markers and positive for CD44, CD73, CD90 and CD105 mesenchymal surface markers. Blue and red clouds represent unstained cells (control) and stained cells with a specific antibody, respectively. (**b**) From top to bottom: undifferentiated hASCs; adipogenic commitment of hASCs characterized by intracellular lipid vacuoles stained in red using Oil Red O (O.R.O) solution; osteogenic commitment of hASCs showing calcium deposits in red after Alizarin Red S staining; chondrogenic commitment of hASCs characterized by cartilage proteoglycans stained with Alcian Blue. Scale bars: 50 μm.

To characterize cells isolated from adipose tissue, a flow cytometry analysis was performed to verify the expression of specific surface markers. Immunophenotypic profiling confirmed that cells exhibited MSC characteristics, since they were negative for CD34 and CD45, two main hematopoietic markers, and they expressed CD44, CD73, CD90 and CD105, specific markers of MSCs (Figure 1a). Moreover, isolated hASCs showed the typical MSC fibroblast-like shape (Figure 1b), and culturing with the appropriate differentiation medium differentiated them into adipogenic, osteogenic and chondrogenic lineages (Figure 1b).

### 2.2. Effects of Cannabidiol on hASC Number and Viability

To evaluate the potential role of CBD in the modulation of the biological properties of hASCs, we treated them with several CBD concentrations (range: 0.1–10 μM) for 72 h and first analyzed the effects of the molecule on cell number and viability. The cell viability, obtained by calculating the percentage of living cells compared to the total number of counted cells, was comparable between control (CTR) and dimethyl sulfoxide (DMSO)/CBD-treated cells (Table 1), except for cells treated with 10 μM CBD, which showed a statistically significant reduction in their viability (Table 1). For this reason, we excluded the 10 μM concentration of CBD from the following investigations.

**Figure 2:**
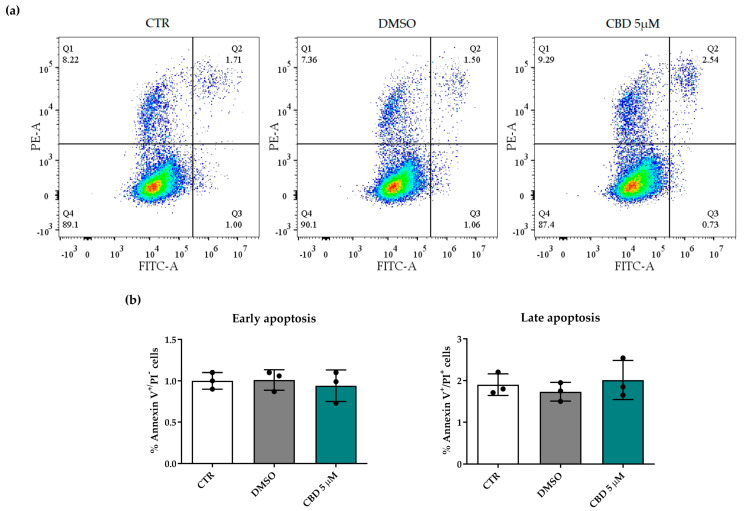
Viability of human adipose-derived stem cells (hASCs) after 72 h of cannabidiol (CBD) treatment. hASC viability was assessed using the annexin V–propidium iodide (PI) staining assay. (**a**) Representative scatter plots of cytofluorimetric analysis for hASCs untreated (CTR) or treated for 72 h with 0.05% dimethyl sulfoxide (DMSO) (CBD vehicle) or with 5 μM CBD. Quadrants (Q) represent the percentage of necrotic cells (Q1), late apoptotic cells (Q2), early apoptotic cells (Q3) and living cells (Q4). FITC-A and PE-A in the scatter plots refer to cells labeled with FITC-conjugated annexin V and PI, respectively. (**b**) Histograms represent the mean percentage of annexin V^+^/PI^−^ (early apoptosis) or annexin V^+^/PI^+^ (late apoptosis) cells ± the standard deviation (SD) in CTR, DMSO and CBD samples (*n* = 3).

To confirm that the CBD 5 µM concentration did not affect cell viability, we further investigated its cytotoxicity with an annexin V–propidium iodide (PI) staining assay. This assay allowed us to distinguish living cells from apoptotic and necrotic cells. Figure 2a shows representative scatter plots of the cytofluorimetric analysis. hASCs treated with 5 µM CBD for 72 h retained high viability comparable to that of the CTR/DMSO samples (Figure 2a, see lower-left quadrant (Q4)). Moreover, CBD-treated hASCs showed a low percentage of annexin V-labeled apoptotic cells (upper- and lower-right quadrants (Q2 and Q3, respectively)) and a similar percentage of PI-labeled necrotic cells (upper-left quadrant (Q1)) compared to CTR and DMSO-treated cells (Figure 2a). As shown in Figure 2b, there were no differences in the percentage of apoptotic cells (in either early or late apoptosis) between CTR, DMSO and CBD samples, confirming that CBD was not toxic for hASCs at the selected concentration (Figure 2b).

### 2.3. Effects of Cannabidiol on hASC Proliferation and Metabolism

**Figure 3:**
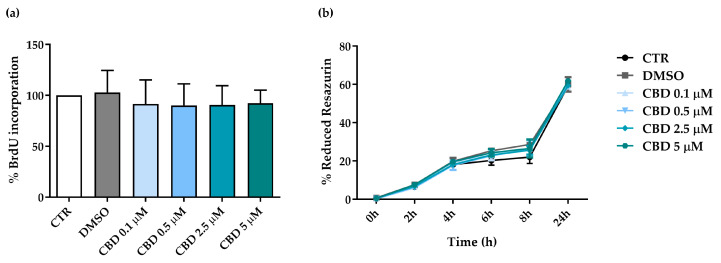
Evaluation of proliferative ability and metabolic activity in human adipose-derived stem cells (hASCs) after 72 h of treatment with cannabidiol (CBD). (**a**,**b**) hASCs were untreated (CTR) or treated with dimethyl sulfoxide (0.05% DMSO, the CBD vehicle) or CBD at concentrations of 0.1, 0.5, 2.5 and 5 µM. (**a**) Histograms represent the mean percentage of bromodeoxyuridine (BrdU) incorporation normalized to CTR ± standard deviation (SD). Analysis was performed after 72 h of treatment (*n* = 3). (**b**) After 48 h from the beginning of treatment, 20 µL of resazurin was added to each sample, and its reduction was measured at 0, 2, 4, 6, 8 and 24 h (corresponding to a 72 h- treatment). Data are expressed as mean percentages of reduced resazurin ± standard deviation (SD) (*n* = 3).

To study the effects of CBD on cell proliferation, hASCs were incubated with a 5-Bromo-2′-deoxyuridine (BrdU) labeling solution following a 72 h-treatment with CBD. BrdU, a thymidine analog, was incorporated in the newly synthesized DNA strands in dividing cells. CBD did not affect hASC proliferation compared to CTR or DMSO-treated cells (Figure 3a). A similar trend was also observed for the metabolic activity of hASCs (Figure 3b).

### 2.4. Effects of Cannabidiol on Spontaneous Adipogenic Commitment in hASCs

#### 2.4.1. Oil Red O (O.R.O) Staining and Lipid Droplet Analysis

To assess the effect of CBD on the adipogenic potential of hASCs, cells were cultured for 14 days in SM alone (CTR) in the presence of CBD at all investigated concentrations or in the presence of 0.05% DMSO. All samples were stained with the O.R.O solution, which colored the lipid vacuoles of differentiated cells red. Data revealed that CBD alone, in the absence of IM, induced a dose-dependent adipogenic commitment of hASCs (Figure S1).

**Figure 4:**
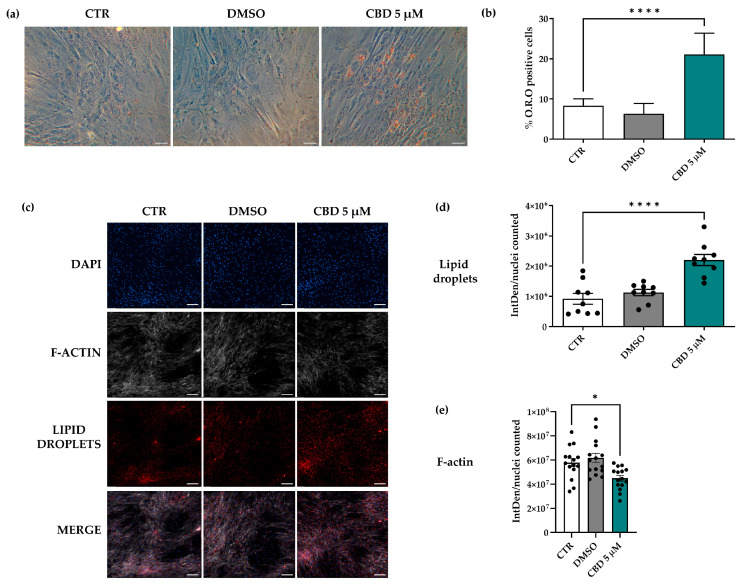
Evaluation of adipogenic potential of human adipose-derived stem cells (hASCs) treated with 5 μM cannabidiol (CBD) for 14 days. (**a**–**e**) hASCs were cultured for 14 days in standard medium (SM) in the absence (CTR) or in the presence of 5 µM CBD or 0.05% dimethyl sulfoxide (DMSO) (CBD vehicle). (**a**) Representative Oil Red O (O.R.O) staining images acquired using a Leica MC170 HD camera. Differentiated hASCs showed red-stained lipid vacuoles in the cytoplasm. Scale bars: 50 μm. (**b**) Histograms represent the mean percentage of O.R.O-positive cells manually counted ± standard deviation (SD); *n* = 3; **** *p* < 0.0001 vs. CTR. (**c**–**e**) Fluorescence analysis of lipid droplets and F-actin in hASCs. (**c**) Lipid droplet content (BODIPY, red signal) and actin expression (phalloidin, grey signal). Nuclei were stained with NucBlue^TM^ (DAPI, blue signal). Images were acquired by a Nikon Eclipse Ti2-E inverted microscope (Nikon Instruments, Melville, NY, USA) and a DS-Qi2 digital sight camera using NIS-Elements imaging software advanced research version 5. (**d**,**e**) Histograms represent the means of Integrated Density (IntDen)/counted nuclei ± standard error of the mean (SEM); *n* = 3; * *p* < 0.05 and **** *p* < 0.0001 vs. CTR.

Moreover, the count of O.R.O-positive cells demonstrated that adipogenesis was significantly increased only in hASCs treated with 2.5 and 5 µM CBD compared to CTR cells (Figure S1 and Figure 4a,b). Since 5 μM CBD was non-toxic and showed the highest efficacy in promoting adipogenesis in hASCs, all subsequent experiments were conducted only using this concentration.

The role of CBD in hASC adipogenesis was confirmed by the quantification of the integrated density in the fluorescence analysis, which revealed a significant increase in lipid droplets in CBD-treated hASCs compared to CTR cells (Figure 4c,d). A similar analysis showed that CBD treatment also significantly reduced the presence of F-actin (Figure 4c,e). For all considered parameters, DMSO-treated cells did not show significant differences relative to CTR cells (Figure 4). This result highlighted that the adipogenic effect of CBD on hASCs was due exclusively to this molecule and was not mediated by DMSO, the CBD vehicle.

#### 2.4.2. Gene Expression Analysis of Adipogenic and Osteogenic Markers

**Figure 5:**
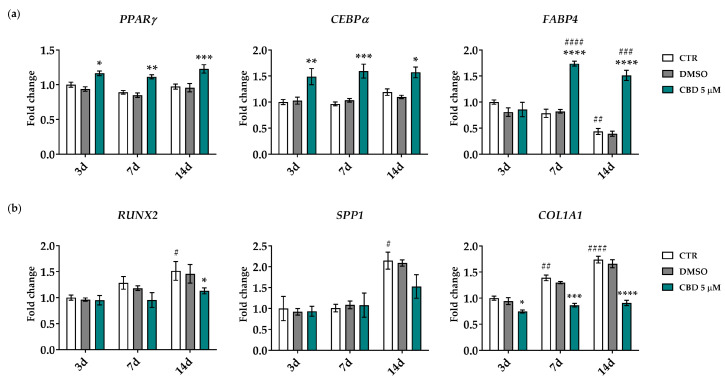
Gene expression analysis of adipogenic and osteogenic markers 3, 7 and 14 days from the beginning of the cannabidiol (CBD) treatment. *Peroxisome proliferator-activated receptor gamma* (*PPARγ*), *CCAAT enhancer-binding protein alpha* (*CEBPα*) and *fatty acid-binding protein 4* (*FABP4*) adipogenic genes (**a**) and *runt-related transcription factor 2 (RUNX2)*, *secreted phosphoprotein 1* (*SPP1*) and *collagen type I alpha 1 chain* (*COL1A1*) osteogenic genes (**b**) were analyzed. (**a**,**b**) Data were normalized using three reference genes (*TATA box binding protein*-*TBP*, *glyceraldehyde-3-phosphate dehydrogenase*-*GAPDH* and *hypoxanthine phosphoribosyltransferase 1*-*HPRT1*); the normalized expression value of CTR after 3 days (white column, 3d) was set to 1, and all other gene expression values are reported relative to that sample. Data are reported as normalized fold change ± standard error of the mean (SEM); *n* = 3; * *p* < 0.05, ** *p* < 0.01, *** *p* < 0.001 and **** *p* < 0.0001: CBD-treated cells vs. CTR cells at the same experimental time points; # *p* < 0.05, ## *p* < 0.01, ### *p* < 0.001 and #### *p* < 0.0001: CTR and CBD-treated cells vs. corresponding samples on day 3. Statistical significance is not shown for DMSO-treated cells, while their behavior was similar to that of CTR cells.

To confirm the adipogenic differentiation results described above, we analyzed the gene expression of adipogenic and osteogenic markers, as these commitments are known to usually proceed in opposite directions. The gene expression of three adipogenic marker genes (*PPARγ, CEBPα* and *FABP4*) was investigated 3, 7 and 14 days from the beginning of CBD treatment. The early *PPARγ* and *CEBPα* markers were statistically upregulated in CBD-treated cells from day 3, while late marker *FABP4* was statistically upregulated from day 7 of treatment compared to the same experimental time point of CTR cells (Figure 5a). Furthermore, in CBD-treated cells, the increased expression of *PPARγ* and *CEBPα* remained stable throughout the differentiation process. Instead, the expression of *FABP4* significantly increased over time, starting from day 7 of treatment (Figure 5a). The expression of these genes in DMSO-treated cells was similar to that of CTR cells at all experimental points and over time, except for *FABP4*, which was statistically downregulated on day 14 with respect to day 3 in both CTR and DMSO samples (Figure 5a).

The expression of osteogenic marker genes *runt-related transcription factor 2* (*RUNX2*) and *secreted phosphoprotein 1* (*SPP1*, also known as osteopontin) 14 days from the start of treatment was lower in CBD-treated cells compared to CTR cells at the same experimental time points; this decrease reached statistical significance only for *RUNX2* (Figure 5b). A significant downregulation was also observed for *collagen type I alpha 1 chain* (*COL1A1*) gene expression starting from day 3 of treatment (Figure 5b). Moreover, the expression of all three genes remained stable throughout the differentiation process in CBD-treated cells. In contrast, their expression increased with statistical significance in CTR cells from day 14 and day 7 for *RUNX2*/*SPP1* and *COL1A1*, respectively. The expression of these genes in DMSO-treated cells was similar to that in CTR cells (Figure 5b).

#### 2.4.3. Spontaneous and Induced Adipogenic Commitment: Morphology and Dimension of Lipid Droplets in hASCs

**Figure 6:**
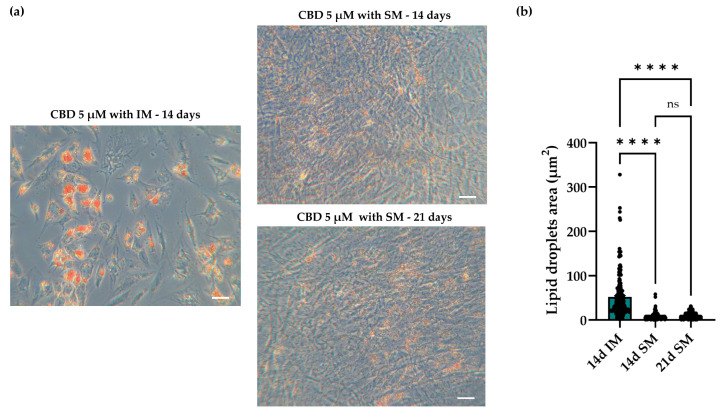
Morphology of human adipose-derived stem cells (hASCs) treated with cannabidiol (CBD) and cultured in the presence or absence of the adipogenic induction medium (IM). (**a**) Representative images of Oil Red O (O.R.O) staining acquired using a Leica Labovert FS inverted microscope equipped with a Leica MC170 HD Imaging System camera. SM: standard medium; 14 and 21 days (14 d and 21 d): times of CBD treatment. Scale bars: 50 μm. (**b**) Analysis of the dimension of lipid droplets. Dots represent the number of droplets analyzed. Data are presented as mean of the lipid droplet area ± standard error of the mean (SEM). ns: not significant; **** *p* < 0.0001.

Finally, we evaluate the differences in the adipogenic process in hASCs treated with 5 μM CBD and cultured in SM or in adipogenic IM. The analysis of cell morphology, assessing outcomes 14 days after the start of the differentiation protocol, showed that hASCs cultured in SM had a fibroblast-like shape, while hASCs cultured in the presence of IM changed their shape and became more enlarged and rounded (Figure 6a). Moreover, hASCs treated with CBD and cultured in IM showed vacuoles with larger lipid droplets compared to CBD-treated cells cultured in SM (Figure 6a,b).

To evaluate whether a prolonged exposure to CBD might improve the differentiation process, hASCs were treated for 21 days with 5 μM CBD in SM. In this condition, the shape of hASCs and the size of the lipid droplets did not differ with respect to cells treated for 14 days; these results indicate that CBD alone was not able to induce a complete differentiation program under our experimental conditions; differentiation program that instead completely occurs in the presence of IM (Figure 6).

## 3. Discussion

In recent years, SC-based therapies have represented promising therapeutic tools for the regeneration and repair of organs and tissues compromised by various pathologies [21,22]. Among the different sources of MSCs, hASCs have assumed an important role in this field due to their high proliferation potential and ability to differentiate into various cell lineages [23,24]. Several studies have shown that these cells promote tissue regeneration, participating in anti-fibrotic, anti-apoptotic and anti-inflammatory actions [24,25]. The ability of hASCs to differentiate into several lineages is crucial for regenerative medicine approaches, especially in clinical applications based on subcutaneous adipose-layer reconstruction [5]. For example, in the field of facial and breast plastic surgery, ASCs are used in autologous free-fat grafting (AFG) to counteract the resorption of grafted tissue commonly linked to this technique. This approach exploits the multifaceted ability of ASCs to provide paracrine growth factors as stimuli for the engrafted fat, acting as natural biological scaffolds to exert immunomodulatory effects in the host microenvironment, as well as to differentiate into mature adipocytes into the target tissue [26].

A better understanding of the molecular mechanisms regulating adipogenesis and the identification of new molecules capable of modulating this process both in vitro and in vivo could significantly improve the therapeutic efficacy of hASCs in the field of regenerative medicine [5].

CBD, a compound isolated from *Cannabis sativa* L., has attracted growing interest in recent years due to its safety profile and therapeutic effects, especially in the neurological field [27,28]. Its effect on the proliferation, migration and differentiation of MSCs isolated from different body areas has also been investigated [14,16,18]. However, the role of CBD in adipogenic differentiation has been addressed only in a few studies, prompting us to deepen our understanding of its role in hASCs.

First, our results show that hASCs treated with CBD at different concentrations (range 0.1–10 µM) showed a reduction in cell viability only with the highest investigated concentration (CBD 10 µM). A similar result was observed by Yu and co-workers when they treated SCs obtained from dental pulp (DPSCs) with 12.5 µM CBD [19]. In contrast with our findings, other researchers did not observe a reduction in viability in hASCs treated with 10 µM CBD; this discrepancy could be explained by the different duration of CBD treatment (24 h) [20]. Moreover, our data demonstrate that CBD concentrations ranging from 0.1 to 5 µM showed high levels of viability comparable to untreated (CTR) cells. These findings are similar to those reported by Li and colleagues in bone marrow-derived MSCs (BM-MSCs) [29] and are not in contrast with the increased viability observed in other studies [19,20].

Moreover, our data revealed that CBD did not influence the cell number, the proliferative ability or the metabolic activity of hASCs. On the contrary, Fellous and colleagues described an increase in the number of BM-MSCs after treatment with 5 μM CBD [16]; such a discrepancy could be due to the different cell model used in their research.

Next, we describe the ability of CBD to commit hASCs towards an adipogenic lineage. Previous studies have shown that CBD can positively influence this process in human and murine ASCs, as well as in murine BM-MSCs, as demonstrated by the increased expression of adipogenic markers CEBPα and FABP4 and the formation of lipid vacuoles in SC cytoplasm [16,18]. In all these studies, MSCs were cultured in the presence of the adipogenic IM, composed of several molecules, including insulin, dexamethasone, 8-methoxymethyl-3-isobutyl-1-methylxanthine (IBMX) and rosiglitazone [30]. Insulin induces proliferation and differentiation of pre-adipocytes [31], and dexamethasone stimulates adipogenic differentiation by increasing the number of adipocytes [32], while IBMX, in the presence of dexamethasone, regulates the expression of PPARγ [33] and consequently promotes the activation of CEBPδ and CEBPβ, two regulators of growth and differentiation [34]. Finally, rosiglitazone promotes lipid accumulation and the formation of large vacuoles [35].

Interestingly, our results demonstrate that CBD, in the absence of IM, promoted adipogenesis in an hASC cell model. In fact, cells treated with CBD for up to 14 days showed spontaneous formation of lipid vacuoles in their cytoplasm, highlighted by O.R.O staining and BODIPY analysis. This effect was dose-dependent and was confirmed by the significant increase in lipid droplet contents in 5 µM CBD-treated hASCs compared to CTR and DMSO-treated cells. This result highlights that the adipogenic effect on hASCs was due exclusively to CBD and was not mediated by DMSO, which was used as a CBD vehicle. However, the morphology of CBD-treated cells and the size of their lipid droplets differed from those of the cells induced to differentiate in adipogenic IM. hASCs treated with CBD remained more elongated, and the droplets were significantly smaller than CBD-treated cells in adipogenic IM. The difference in droplets size may be due to the chemical characteristics of the CBD, with its acidic form preventing an increase in droplet dimension, unlike the rosiglitazone contained in the IM formulation, which, as described above [35], promotes the formation of larger vacuoles. Overall, the emergent differences could be justified by the fact that CBD alone cannot reproduce the effects exerted by the multiple molecules included in the IM. In addition, the same droplet size and morphology found in hASCs treated with CBD for 14 days were observed when the treatment was extended to 21 days, confirming a different action of CBD than IM in inducing adipogenesis. On the other hand, the intrinsic pro-adipogenic activity of CBD could have several advantages in SC-based approaches to regenerative medicine. For example, CBD, being a natural molecule, could be easy to use in vivo, either alone or in combination with other molecules, resulting in fewer side effects than synthetic substances that are present in adipogenic IM.

In our study, adipogenesis in cells treated with 5 μM CBD was also confirmed by the increased expression of adipogenic markers *PPARγ*, *CEBPα* and *FABP4* compared to CTR cells starting from precise experimental time points. Almousa and colleagues observed an increase in *PPARγ* expression and a decrease in the *CEBPα* level in hMSCs cultured in SM with CBD. However, in their study, the treatment was carried out with 1 μM CBD for only 72 h, and the type of MSCs was not specified, justifying a different trend in gene expression [35]. The ability of hASCs to spontaneously differentiate has already been reported in other studies that used different culture conditions or cells at different culture passages than those used in our research. For example, a study by Romaldini and colleagues reported increased expression levels of *CEBPα* and *PPARγ* in hASCs cultured in SM supplemented with 10% human serum rather than with 10% FBS [36]. In another study conducted by Yang and colleagues, an increase in the expression of the same genes was observed in murine ASCs at the first culture passage compared to those of subsequent passages (2 to 5). This suggests a decline in the potential for spontaneous adipogenic differentiation as cell culture passages progress [37]. The fact that, in our study, hASCs at the third through sixth culture passages, treated with 5 µM CBD, still showed the ability to differentiate into adipocytes in the absence of IM underlines the potential of CBD in modulating the adipogenic process.

Several studies have reported that CBD, when added to osteogenic IM, acts as an inducer of MSC osteoblastic differentiation, promoting alkaline phosphatase activity (ALP) and bone mineralization associated with the expression of osteogenic markers such as RUNX2, Osterix, Osteocalcin (OCN) and COL1A1 [14,38,39]. However, its role in the modulation of osteogenesis in the absence of IM remains unexplored. In our study, the stability in the expression of osteogenic markers *RUNX2*, *SPP1* and *COL1A1* during 14-day exposure to CBD and their decreased expression after 7 and/or 14 days compared to corresponding CTR cells, as well as the reduced presence of actin after 14 days of CBD treatment, could reinforce the hypothesis of a pro-adipogenic role of CBD. It is known that adipogenesis and osteogenesis processes in MSCs depend on specific changes in mechanical properties and molecular pathways [40,41]. Several studies have demonstrated that a less organized actin cytoskeleton favors adipogenesis [42], as observed in CBD-treated cells, whereas osteogenesis requires larger numbers of focal adhesions and a stiff, spread actin cytoskeleton [42,43]. In addition, molecular pathways that guide adipogenesis and osteogenesis are often regulated in an opposed fashion. Thus, when the expression of adipogenic markers increases, the levels of osteogenic markers tend to decrease [44,45], as we demonstrated in our model.

## 4. Materials and Methods

### 4.1. Harvesting and Culturing of hASCs

hASCs were isolated from the adipose tissue of healthy female subjects upon signing of the informed consent. hASCs were harvested as described in our previously published study [46]. For experiments, hASCs were seeded at different densities in adequate plastic supports (Corning Incorporated, Corning, NY, USA) as specified in the following sections; cells were incubated under standard conditions for 24 h before treatment. Experiments were performed using hASCs in the 3rd–6th culture passages and repeated in biological triplicate (*n* = 3).

### 4.2. hASC Characterization

To characterize the cells after their harvesting, the expression of specific surface markers was evaluated using a CytoFLEX S flow cytometer (Beckman-Coulter Inc., Brea, CA, USA). For this purpose, monoclonal antibodies (ABs) for mesenchymal surface markers conjugated with fluorescein isothiocyanate (FITC) (CD44, CD90) or phycoerythrin (PE) (CD105 and CD73) and for hematopoietic markers conjugated with peridinin chlorophyll (PerCP) (CD34, CD45) (eBioscienceTM, Thermo Fisher Scientific, San Diego, CA, USA) were used. Data acquisition and analysis were performed using FlowJo v10.8 software (Tree Star, Ashland, OR, USA), following the protocol previously described in [47]. hASC trilineage differentiation potential was assessed by using specific stainings. The adipogenic potential was evaluated as described in Section 4.8. The osteogenic and chondrogenic potentials of hASCs were assessed by seeding cells at a density of 5000 cells/cm^2^ in a 24-well plate. Once cells reached 70% and 90% confluence, osteogenesis and chondrogenesis, respectively, were induced as previously described [48], following the manufacturer’s recommendations.

### 4.3. Cannabidiol Treatments

Cannabidiol (CBD) (Farmech Società Agricola SRL, Milan, Italy) was dissolved in dimethyl sulfoxide (DMSO) at a final concentration of 3 mg/mL, then diluted in complete medium at different concentrations (0.1, 0.5, 2.5, 5 and 10 µM). A volume of 10 µM CBD was used for preliminary investigations on cell viability. Due to the increased cytotoxicity observed in cells treated with CBD at this concentration, we excluded them from all the following assays. Both cells treated with 0.05% DMSO (the CBD vehicle) and untreated cells (CTR) were used as controls.

### 4.4. Cell Count and Viability

The hASCs were seeded in a 6-well plate at a density of 5000 cells/cm^2^ and kept in the culture medium (CTR) or treated with CBD (range 0.1–10 μM) or with 0.05% DMSO for 72 h. For each sample, viable (unstained cells) and dead cells (cells stained red with Erythrosine B dye 0.2%, Sigma-Aldrich Co., St. Louis, MO, USA) were counted as previously described [47]. Cell viability was expressed as the percentage of living cells compared to the total cell number ± standard deviation (SD).

### 4.5. Annexin V Apoptosis Detection Assay

hASCs were seeded in 75 cm^2^ flasks at a density of 3500 cells/cm^2^ and unexposed or exposed to 5 μM CBD or 0.05% DMSO for 72 h. A commercial eBioscience^TM^ Annexin V Apoptosis Detection Kit with PI (Thermo Fisher Scientific, Waltham, MA, USA) was used to evaluate the viability and the death of hASCs after CBD treatment, and the manufacturer’s instructions were followed as previously reported [47]. Briefly, cells were initially incubated with annexin V conjugated to FITC-A (5 μL) to detect apoptotic cells; then, after a wash with a provided buffer, PI was added to the cell suspension to a final concentration of 20 μg/mL (10 μL) to identify necrotic cells. Fluorescence was acquired using a CytoFLEX S flow cytometer (Beckman-Coulter Inc., Brea, CA, USA), and data were analyzed using FlowJo v10.8 software (Tree Star, Ashland, OR, USA). In scatter plots, the lower-left quadrant (Q4) indicates living cells (which are negative for both annexin V and PI markers); the lower-right quadrant (Q3) shows hASCs in the early stage of apoptosis (labeled as FITC-conjugated annexin V); the upper-left quadrant (Q1) underlines cells in necrosis (labeled as PI); and the upper-right quadrant (Q2) highlights cells in the late stage of apoptosis, which are positive for both markers. Results are reported as mean percentages of apoptotic (both early and late apoptosis) cells ± SD.

### 4.6. Bromodeoxyuridine (BrdU) Assay

hASCs, seeded on a 96-well plate at the density of 6000 cells/cm^2^ (3 wells/condition), were untreated or treated for 72 h with CBD (range 0.1–5 μM) or 0.05% DMSO. At the end of the treatment, a BrdU assay (Roche, Basel, Switzerland) was performed, following the manufacturer’s instructions as previously reported [47]. BrdU, an analog of thymine incorporated into the DNA of proliferating cells, was quantified using a peroxidase-conjugated anti-BrdU antibody. The absorbance at 450 nm was measured with a Wallac 1420 Victor^2^ Multilabel Counter (Perkin Elmer, Waltham, MA, USA). Cell proliferative ability was expressed as a mean percentage of BrdU incorporated in treated cells compared to CTR cells (set to 100%) ± SD.

### 4.7. Resazurin-Based Assay

To assess hASC metabolism, cells were seeded in a 96-well plate at a density of 4000 cells/cm^2^ (3 wells/condition), and an “AlamarBlue Cell Viability assay” (Invitrogen, ThermoFisher Scientific, Waltham, MA, USA) was used [49]. After 48 h from the beginning of treatment with CBD (range 0.1–5 μM) or 0.05% DMSO, as well as in untreated cells, 20 µL of resazurin was added to each sample, and its reduction by metabolically active cells was measured at 0, 2, 4, 6, 8 and 24 h (corresponding to 72 h of treatment). Fluorescence acquisition and data analysis followed the methods reported in a previous manuscript [48]. hASC metabolism was expressed as percentage of resazurin reduction according to the following formula: (FI 590 of test agent − FI 590 of negative control)/(FI 590 of 100% reduced resazurin − FI 590 negative control) × 100, where FI is fluorescence intensity.

### 4.8. O.R.O Staining and Analysis of Lipid Droplet Size

To assess the effect of CBD on hASC adipogenic differentiation capability, cells were seeded in a 48-well plate at a density of 9000 cells/cm^2^ (2 wells/condition). hASCs at 100% confluence were cultured in SM in the presence of CBD (0.1, 0.5, 2.5 and 5 μM) or 0.05% DMSO. Untreated cells were used as CTR. Media were changed twice a week for 14 days. After 14 days, O.R.O staining (Sigma Aldrich Co., St. Louis, MO, USA) and the subsequent image acquisition were performed as described in a previous manuscript [48]. Unstained (undifferentiated) and stained (differentiated) cells were counted using ImageJ software 1.53 [50]. Data are reported as the means of the percentage of O.R.O-positive cells ± SD.

In addition, hASCs were cultured for 14 days with both 5 μM CBD and StemPro Adipogenesis Differentiation medium (IM-Thermo Fisher Scientific, Waltham, MA, USA) to evaluate whether IM induced morphological differences in these cells compared to those treated with only 5 μM CBD for 14 or 21 days. For each condition, 5 images were acquired, and the size of red lipid droplets (at least 200 for each condition) was calculated using ImageJ 1.53 software. The results are presented as the mean of the area of lipid droplets (μm^2^) ± standard error of the mean (SEM).

### 4.9. Staining and Quantification of Lipid Droplets and Actin

hASCs were seeded in a 24-well plate containing glass coverslips (Waldemar Knittel Glasbearbeitungs GmbH, Braunschweig, Germany) at a density of 9000 cells/cm^2^ and treated for 14 days with 5 µM CBD or 0.05% DMSO in complete medium. Untreated cells were used as a CTR. Each experimental condition was performed in technical duplicate. At the end of treatment, cells were fixed with 4% formaldehyde (Sigma-Aldrich Co., St. Louis, MO, USA) for 15 min and washed with Phosphate-Buffered Saline 1X (PBS 1X, Euroclone, Pero, MI, Italy). To visualize lipid droplets and F-actin, cells were incubated with BODIPY™ TR Ceramide red fluorescent dye (Thermo Fisher Scientific, Waltham, MA, USA) (dilution 1:400) and Phalloidin-FITC-labelled (P5282; Sigma-Aldrich Co., St. Louis, MO, USA), respectively, for 1 h at room temperature. Incubation with NucBlue^®^ Fixed Cell ReadyProbes^®^ Reagent (DAPI, Molecular Probes™, Life Technologies—Thermo-Fisher Scientific, Waltham, MA, USA) was performed to counter-stain nuclei. All slides were mounted with antifade AF-400 (Immunological Sciences, Rome, Italy). The detection and acquisition of images were performed using a Nikon Eclipse Ti2-E inverted microscope (Nikon Instruments, Melville, NY, USA) and a DS-Qi2 digital sight camera (Nikon Instruments, Melville, NY, USA) through NIS-Elements imaging software advanced research version 5 (from 3 to 5 fields/condition). The fluorescence intensity was quantified by Fiji ImageJ software 2.1.0/1.53c [46,51] and indicated as Integrated Density (IntDen), which refers to the average value of fluorescence in a selected area normalized to the number of cells/nuclei. Data are presented as the means of IntDen ± SEM.

### 4.10. Gene Expression Analysis

#### 4.10.1. RNA Extraction and RT-PCR

To evaluate the expression of genes involved in adipogenic (*PPARγ*, *CEBPα* and *FABP4*) and osteogenic (*RUNX2, SPP1* and *COL1A1*) commitment, hASCs seeded in a 6-well plate at a density of 9000 cells/cm^2^ were harvested for RNA isolation after treatment with 5 µM CBD or 0.05% DMSO. Untreated cells were used as CTR.

Total RNA was extracted 3, 7 and 14 days from the beginning of the treatment using a RNeasy Mini Kit (QIAGEN, Valencia, CA, USA) and digested with RNase-free Deoxyribonuclease I (RNase-free DNase set-QIAGEN, Valencia, CA, USA) following the manufacturer’s instructions. Then, to exclude DNA contamination, the extracted RNA was tested by performing a PCR analysis using a pair of primer sequences that recognize the HSP70 promoter region. PCR products were visualized on 2% agarose gel (Bio-Rad Laboratories, Inc., Hercules, CA, USA) after electrophoresis: the absence of bands in the RNA samples demonstrated that no DNA contamination was present in the investigated samples. Finally, RNA was reverse-transcribed in cDNA (iScript™ RT Supermix; Bio-Rad Laboratories, Inc., Hercules, CA, USA). To verify whether the retrotranscription reaction was successful, *glyceraldehyde 3-phosphate dehydrogenase* (*GAPDH*) gene amplification and amplicon detection were performed as previously described [52].

#### 4.10.2. Real-Time PCR

For qPCR analysis, 25 ng of cDNA was amplified using SsoAdvanced Universal SYBR Green Supermix (Bio-Rad Laboratories, Hercules, CA, USA) in technical triplicates. The reaction was carried out using a Bio-Rad CFX96 real-time thermocycler (Bio-Rad Laboratories, Hercules, CA, USA) with the following protocol: 30 s (sec) at 95 °C, 5 s at 95 °C and 25 s at 60 °C (45 cycles). Gene expression analysis was carried out using CFX Manager Software version 3.1 (Bio-Rad Laboratories, Hercules, CA, USA) using the “delta-delta CT method” [53,54]. For each experimental condition, the expression and the stability of reference genes (*GAPDH*, *TATA box binding protein-TBP* and *hypoxanthine phosphoribosyltransferase 1—HPRT1*) were tested according to the protocol described by Hellemans and collaborators [55]. *GAPDH*, *TBP* and *HPRT1* primers were purchased from Bio-Rad (20X, Bio-Rad Laboratories, Hercules, CA, USA); *HSP70 promoter, CEBPα*, *PPARγ*, *FABP4, RUNX2* and *SPP1* were provided by Sigma-Aldrich (Sigma-Aldrich Co., St. Louis, MO, USA); and *COL1A1* was purchased from Invitrogen (Invitrogen, ThermoFisher Scientific, Waltham, MA, USA). A list of primer sequences is reported in Table 2. The specificity of real-time products was confirmed by analyzing the melting curve of each primer pair [56]. For each investigated gene, the normalized expression value of the CTR cells 3 days from the beginning of the treatment was set to 1, and all the other gene expression values are reported relative to that value. Data are shown as normalized fold change ± SEM.

### 4.11. Statistical Analysis

Statistical analysis was conducted using GraphPad Prism software version 10 (GraphPad Software, San Diego, CA, USA). Appropriate parametric (one-way or two-way ANOVA and post hoc Tukey test) or nonparametric tests (Kruskal–Wallis test with post hoc Dunn’s test) were performed. Results are shown as mean ± SD or mean ± SEM. A *p*-value < 0.05 was considered statistically significant.

## 5. Conclusions

In conclusion, our study provides compelling evidence that CBD plays a pivotal role in modulating the spontaneous adipogenic differentiation of hASCs. The observed effects of CBD, including the formation of lipid vacuoles and increased expression of essential adipogenic markers, suggest that this phytocannabinoid could serve as a promising therapeutic agent in regenerative medicine. Our findings highlight the potential of CBD not only to enhance the adipogenic capacity of hASCs but also to innovate approaches in SC therapies aimed at addressing adipose tissue dysfunctions. Therefore, in the future, it will be important to confirm the effects of CBD on adipogenesis, even in vivo, where its use could offer advantages, being a natural molecule with less adverse/toxic effects compared to synthetic molecules.

Further research will be also necessary to fully elucidate the mechanisms through which CBD exerts its effects on hASCs. Detailed studies exploring the molecular pathways activated by CBD will be crucial for a deeper understanding of its role in adipogenesis. Ultimately, this knowledge may pave the way for the development of more effective regenerative therapies, particularly for conditions characterized by adipose tissue deficits. Therefore, as the field of regenerative medicine continues to evolve, the integration of CBD’s properties into therapeutic strategies could hold significant promise in terms of enhancing tissue repair and recovery, offering novel strategies for optimizing healing outcomes and improving patient well-being.

## Figures and Tables

**Table 1:** Viability of human adipose-derived stem cells (hASCs) after 72 h of cannabidiol (CBD) treatment.

Sample	% of Living Cells
CTR	96.9 ± 0.4
DMSO	98.2 ± 0.8
CBD 0.1 µM	96.6 ± 0.3
CBD 0.5 µM	95.6 ± 0.8
CBD 2.5 µM	97.0 ± 0.5
CBD 5 µM	97.7 ± 0.9
CBD 10 µM	88.8 ± 0.5 ***

CTR: untreated hASCs; DMSO: hASCs treated with 0.05% dimethyl sulfoxide, the cannabidiol (CBD) vehicle; CBD: hASCs treated with CBD. Data are presented as the mean percentage of living cells ± standard deviation (SD); *n* = 3. *** *p* < 0.001 vs. CTR.

**Table 2:** Primer sequences used to analyze gene expression in human adipose-derived stem cells (hASCs).

Gene	Entrez Gene ID	Left Primer	Right Primer	Bio-Rad Unique Assay ID	A.L. (bp)
*HSP70 (promoter)*	3303	CGCCATGGAGACCAACACCC	GCGGTTCCCTGCTCTCTGTC	-	500
*Glyceraldehyde 3-phosphate dehydrogenase (GAPDH)*	2597	-	-	qHsaCED0038674	117
*TATA box binding protein (TBP)*	6908	-	-	qHsaCID0007122	120
*Hypoxanthine phosphoribosyl* *transferase 1(HPRT1)*	3251	-	-	qHsaCID0016375	90
*Peroxisome proliferator-activated* *receptor gamma (PPARγ)*	5468	TTGCAGTGGGGATGTCTCAT	TTTCCTGTCAAGATCGCCCT	-	208
*CCAAT enhancer binding protein* *Alpha (CEBPα)*	1050	GCAAACTCACCGCTCCAATG	TTCTCTCATGGGGGTCTGCT	-	113
*Fatty acid binding protein 4 (FABP4)*	2167	GATAAACTGGTGGTGGAATGCG	ATGCGAACTTCAGTCCAGGT	-	100
*RUNX family transcription* *factor 2 (RUNX2)*	860	CTCCCTGAACTCTGCACCAA	TAGAGTGGATGGACGGGGAC	-	149
*Secreted phosphoprotein 1 (* *SPP1)*	6696	ACTGATTTTCCCACGGACCT	CTCCTCGCTTTCCATGTGTG	-	192
*Collagen type I alpha 1 chain* *(COL1A1)*	1277	TGAAGGGACACAGAGGTTTCAG	GTAGCACCATCATTTCCACGA	-	193

ID: identification number; A.L. (bp): amplicon length (base pair). Primers purchased from Bio-Rad are of unknown sequence (-) due to the policy of the manufacturer.

## Data Availability

Data are contained within the article and Supplementary Materials.

## Supplementary Materials

The following supporting information can be downloaded at https://www.mdpi.com/article/10.3390/molecules30112367/s1: Figure S1: Evaluation of the spontaneous adipogenic potential of human adipose-derived stem cells (hASCs) in the presence of cannabidiol (CBD). hASCs were cultured for a period of 14 days in complete medium alone (CTR) or in the presence of CBD (0.1 µM, 0.5 µM, 2.5 µM and 5 µM) or 0.05% dimethyl sulfoxide (DMSO) (CBD vehicle). (**a**) Representative images of Oil Red O (O.R.O)-stained cells acquired using a Leica MC170 HD camera. hASCs positive for adipogenesis show red-stained lipid vacuoles in the cytoplasm. Scale bars: 50 µm. (**b**) Histograms representing the mean percentage of O.R.O-positive cells manually counted ± standard deviation (SD); *n* = 3; * *p* < 0.05, **** *p* < 0.0001 vs. CTR.
