# Supplementary figures and images for: Cannabidiol as Modulator of Spontaneous Adipogenesis in Human Adipose-Derived Stem Cells
Source: Molecules. 2025 May 29;30(11):2367. doi: 10.3390/molecules30112367 (PMC12156936; doi:10.3390/molecules30112367)

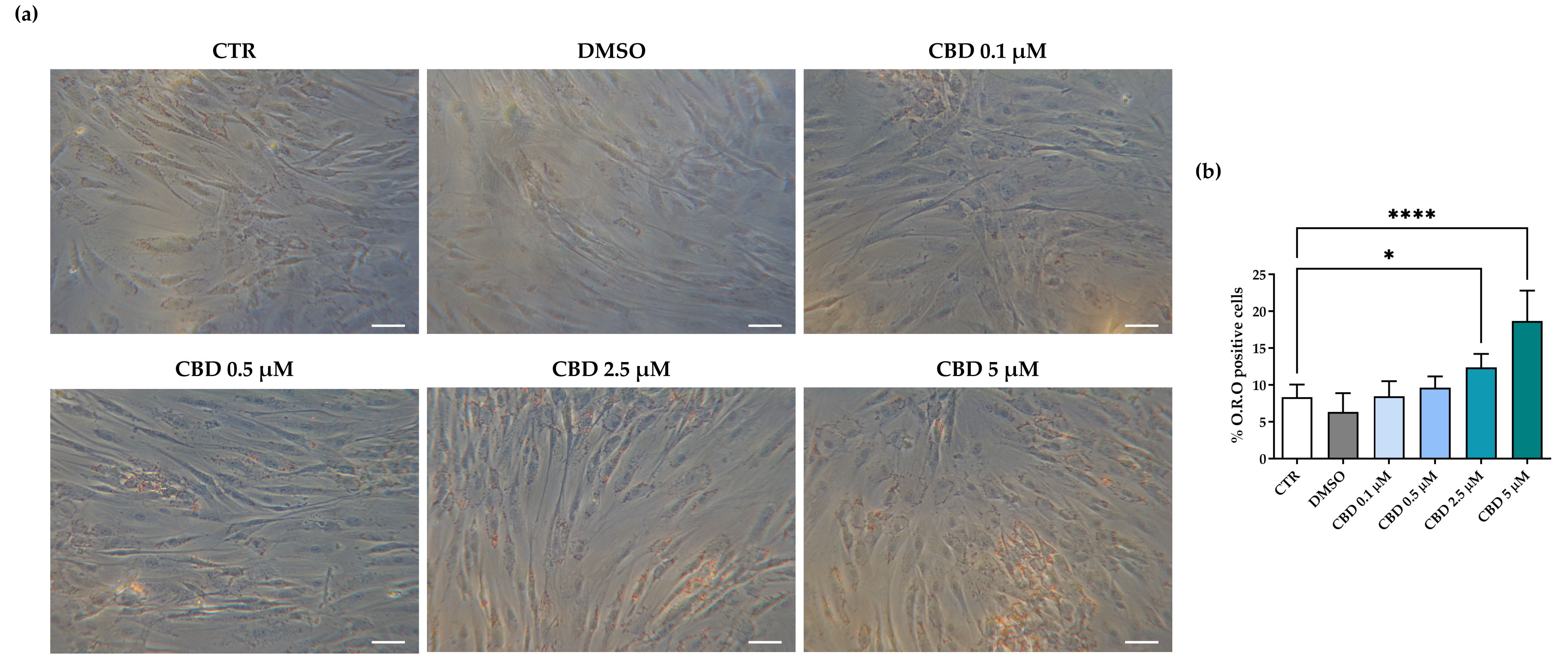

Supplement: Supplementary file 1 [file molecules-30-02367-s001.zip › molecules-3620373-supplementary.jpg]
